# Crocin suppresses multidrug resistance in MRP overexpressing ovarian cancer cell line

**DOI:** 10.1186/s40199-016-0155-8

**Published:** 2016-06-24

**Authors:** Shadi Mahdizadeh, Gholamreza Karimi, Javad Behravan, Sepideh Arabzadeh, Hermann Lage, Fatemeh Kalalinia

**Affiliations:** Department of Cell and Molecular Biology, Kish International Campus, University of Tehran, Kish, Iran; Medical Toxicology Research Center, School of Pharmacy, Mashhad University of Medical Sciences, Mashhad, Iran; Biotechnology Research Center, School of Pharmacy, Mashhad University of Medical Sciences, P. O. Box 91775-1365, Mashhad, Iran; Department of Pharmaceutical Biotechnology, School of Pharmacy, Mashhad University of Medical Sciences, Mashhad, Iran; Institute of Pathology, Charite University, Campus Mitte, Humboldt, Berlin Germany; Medical Genetic Research Center, Mashhad University of Medical Sciences, Mashhad, Iran

**Keywords:** Crocin, Multidrug resistance, MRP1, MRP2, A2780, A2780/RCIS

## Abstract

**Background:**

Crocin, one of the main constituents of saffron extract, has numerous biological effects such as anti-cancer effects. Multidrug resistance-associated proteins 1 and 2 (MRP1 and MRP2) are important elements in the failure of cancer chemotherapy. In this study we aimed to evaluate the effects of crocin on MRP1 and MRP2 expression and function in human ovarian cancer cell line A2780 and its cisplatin-resistant derivative A2780/RCIS cells.

**Methods:**

The cytotoxicity of crocin was assessed by the MTT assay. The effects of crocin on the MRP1 and MRP2 mRNA expression and function were assessed by real-time RT-PCR and MTT assays, respectively.

**Results:**

Our study indicated that crocin reduced cell proliferation in a dose-dependent manner in which the reduction in proliferation rate was more noticeable in the A2780 cell line compared to A2780/RCIS. Crocin reduced MRP1 and MRP2 gene expression at the mRNA level in A2780/RCIS cells. It increased doxorubicin cytotoxicity on the resistant A2780/RCIS cells in comparison with the drug-sensitive A2780 cells.

**Conclusion:**

Totally, these results indicated that crocin could suppress drug resistance via down regulation of MRP transporters in the human ovarian cancer resistant cell line.

## Background

Cancer is a leading cause of death in the world that broadly affects more and less economically developed countries [1]. Using different chemotherapy regimens is a common method in cancer treatment, but there is some limitation on the effectiveness of chemotherapy that leads to poor therapeutic results [2]. One of the most important reasons of treatment failure during chemotherapy is the multidrug resistance (MDR) phenomenon. The MDR tumors are resistant to chemotherapeutic agents which are structurally and functionally different from the initial anticancer drug. Typical MDR occurs through overexpression of the membrane efflux proteins that pump anticancer drugs out of the cells [3]. These pharmaceutical transporter proteins belong to the ATP-binding cassette transporters family (ABC family) [4, 5]. One important class of the ABC family is the human multidrug resistance-associated protein (MRP) family which contains seven members. Several members of the MRP family especially MRP1 and MRP2 are involved in the detoxification and protection of the host against xenobiotic materials. They are also assumed to cause drug resistance through their ability in transporting a wide range of anticancer drugs out of the cells and their presence in many different types of tumors [6].

In the last decades, different research explored new botanical candidates with potential anti-cancer effects that has opened a window to developing safer and more effective anti-cancer therapies [7, 8]. *Crocus sativus* is a plant of the Iridaceae family. Stigmas of *Crocus sativus* flowers (saffron) contain various chemical substances [9]. Crocin is a major glycosylated carotenoid found in saffron [10] that has various pharmacological effects like protecting the myocardial cell against hypoxia damage [11], antioxidant [12, 13], anti-atherosclerosis [14, 15], antidepressant [16] and anti-inflammatory effects [17, 18]. In addition, different studies have shown anticancer activities of crocin against human leukemia, breast, colorectal, and bladder cancer cell lines [19–23]. Based on these facts, it is expected that crocin could potentially be used clinically for the prevention and treatment of cancer in the near future.

It has shown that crocin inhibits Lipopolysaccharides (LPS)-induced nitric oxide (NO) release from brain microglial cells and reduces the LPS-stimulated productions of tumor necrosis factor-alpha, interleukin-1 beta, and intracellular reactive oxygen species, which effectively cause decreased NF-kappa B activation [17, 24]. On the other hand, it has been previously showed that sulindac, the nonsteroidal anti-inflammatory drug, generates oxidative stress via induction of reactive oxygen species (ROS) production, which finally leads to the higher expression of MRP1 and MRP3 in human colorectal cancer cell lines [25]. These evidences suggest that crocin might affect the protein expression of MDR proteins. In the present study, we aimed to evaluate the effects of crocin on the expression and function of MRP1 and MRP2 in the human ovarian carcinoma cell lines A2780 and its cisplatin-resistant derivative A2780/RCIS cells (MRP2-overexpressing cell line).

## Methods

### Materials

Fetal bovine serum (FBS) and RPMI 1640 with L-glutamine were purchased from Gibco (USA) and Biosera (UK), respectively. MTT, DMSO, trypan blue, doxorubicin and penicillin G/streptomycin were obtained from Sigma-Aldrich (Germany). Crocin was generously provided by Dr. Seyed Ahmad Mohajeri (Pharmaceutical Research Center, Mashhad University of Medical Sciences, Iran). RNA tripure isolation kit was obtained from Roche Applied Science, Germany and Real-time EXPRESS One-Step SYBR GreenER™ Kit was purchased from Invitrogen, USA. The MRP-overexpressing, cisplatin-resistant ovarian cancer cell line, A2780/RCIS and its parental cisplatin sensitive cell line A2780 were generously provided by Professor Herman Lage (Molecular Pathology Department, Charite Campus Mitte, Berlin, Germany).

### Preparation of the crocin solution

Total crocin was extracted and crystallized from saffron stigmas and its purity was tested with HPLC and was more than 96 % [26]. Crocin was dissolved in DMSO (dimethyl sulfoxide) and PBS to a final concentration of 1024 mM and stored at −20 °C. The drug was freshly diluted to its final concentration (10, 20, 40, 60, 80 and 100 μM) in culture medium prior to the start of each experiment.

### Cell culture and treatment

Cells were cultured in RPMI-1640 contained FBS 10 % (v/v), penicillin (100 U/mL), and streptomycin (100 μg/mL) at 37 °C in humidified air containing CO2 5 %. For MTT and real-time PCR studies, ovarian cancer cells were incubated for 4–72 h with crocin (0–100 μM). For MRP activity analysis, all cell lines were co-treated with different concentrations of crocin (0–100 μM) and doxorubicin (0–500 nM) for 4–72 h. This study was obtained the approval of the Research Ethics Committee of Mashhad University of Medical Sciences (code No: IR.MUMS.REC.1390.301).

### MTT cytotoxicity assay

Drug sensitivity of the A2780 cell line and drug-resistant cell line A2780/RCIS were confirmed by MTT assay. Cells were seeded at an initial density of 10^4^ cells/well in 96-well plates. The plates were incubated at 37 °C in a 5 % CO2-supplemented atmosphere for 24 h. Subconfluent cells were treated with different concentrations of crocin and doxorubicin in a final volume of 100 μl of standard growth medium in each well. The control wells had DMSO in the growth medium at equal volumes to those used for the test compounds. Cell viability was measured after 4–72 h, using 3-(4, 5-dimethylthiazol-2-yl)-2, 5-diphenyl tetrazolium bromide (MTT). The reduced MTT dye was solubilized with DMSO (100 μl/well) and absorbance was determined on an ELISA plate reader (BioTek, Bad Friedrichshall, Germany) with a test wavelength of 550 nm and a reference wavelength of 630 nm. Each experiment was performed in triplicate and was repeated at least three times. The percentage of cell proliferation was calculated using the ratio of OD_test_/OD_control_.

### Real-Time RT-PCR

Total cellular RNA was extracted using tripure isolation reagent. The total amount of RNA was measured using a NanoDrop 1000 spectrophotometer (Thermo Fisher Scientific, Wilmington, DE) and the acceptable purity was in the range of 1.8-2.2 for the A260/A230 and A260/A280 ratios. Real-time RT-PCR was performed to measure the expression levels of MRP1 and MRP2 in ovarian cancer cell lines using the EXPRESS One-Step SYBR GreenER™ Kit and real-time cycler Mx3000P™ Stratagen (Stratagen, USA). The primers had the following sequences: MRP1: 5´-GTGTTTCTGGTCAGCCCAACT-3´ (forward) and 5´-TTGGATCTCAGGATGGCAGG-3´ (reverse); MRP2, 5′-AGCAGCCATAGAGCTGGCCCTT-3′ (forward) and 5′-AGCAAAACCAGGAGCCATGTGCC-3′ (reverse); β-actin: 5´-TCATGAAGTGTGACGTGGACATC-3´ (forward) and 5´-CAGGAGGAGCAATGATCTTGATCT-3´ (reverse). Reactions were performed with an initial cDNA synthesis step at 50 °C for 5 min, followed by the denaturation step at 95 °C for 2 min and PCR amplification cycles (40 cycles at 95 °C for 15 s, 60 °C for 1 min). Relative expression levels for MRP1 or MRP2 were normalized to the β-actin by the MxPro-Mx3000P system. The relative expressions of MRP genes were reported as the target/reference ratio of the treated samples divided by the target/reference ratio of the untreated control sample.

### Statistical analysis

Results (mean ± SD) were reported in three independent stages. Statistical analyses were performed by SPSS version 16.0 using ANOVA, with the Tukey's post-hoc to show significant differences between the data and *p* values < 0.05 were considered significant.

## Results

### Effect of crocin on the proliferation rate of A2780 cancer cell lines

**Fig. 1:**
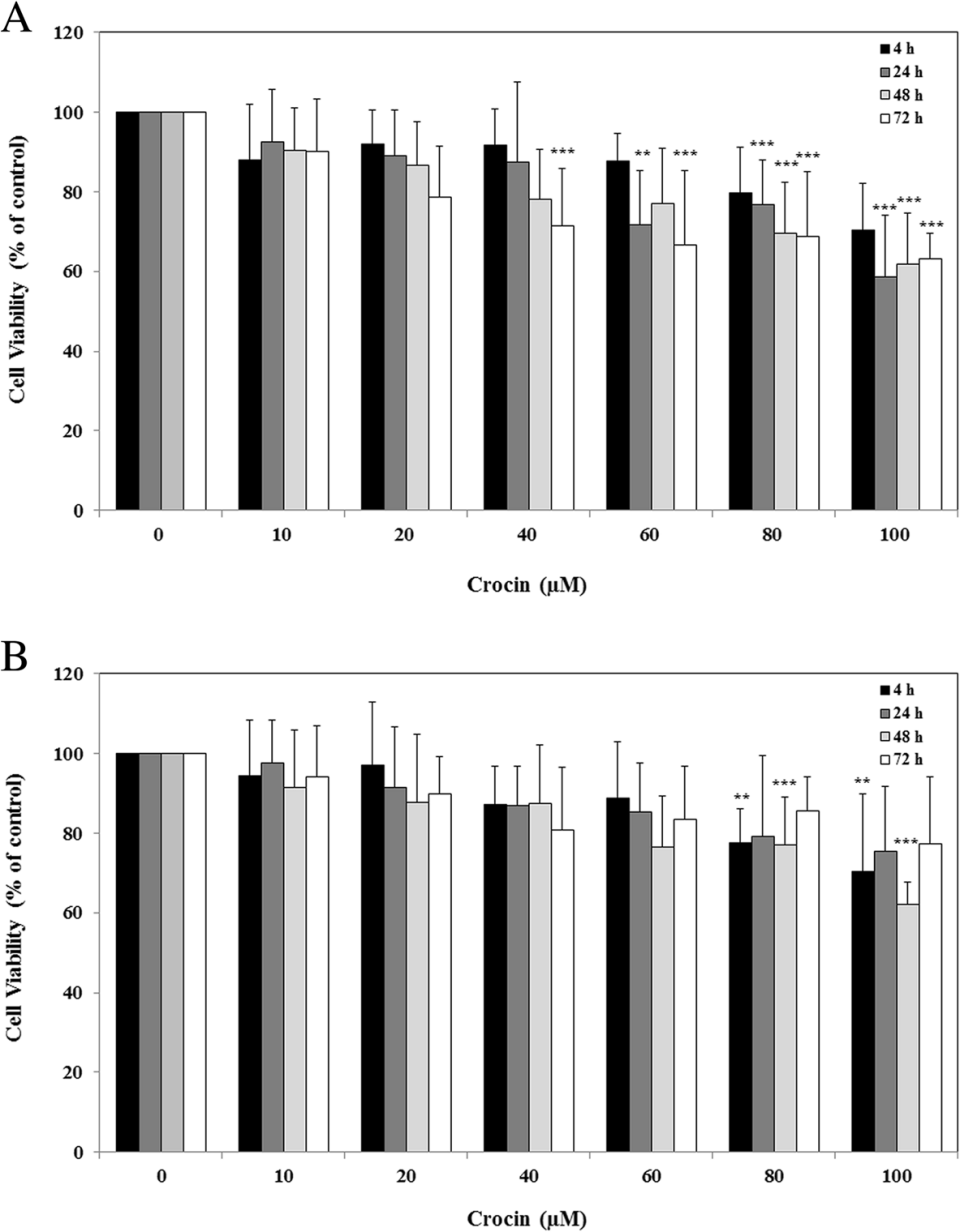
The effects of crocin on cell viability of A2780 (**a**) and A2780/RCIS (**b**) cell lines. The cells were incubated with various concentrations of crocin at 37 °C for 4, 24, 48 and 72 h. Cell viability was measured by the MTT assay. Each experiment was repeated independently three times in triplicate tests and data are shown as mean ± SD. *P ≤ 0.05; **P ≤ 0.01; ***P ≤ 0.001

To investigate the effects of crocin on the cell survival of ovarian cancer cells, A2780 cells were incubated in the presence or absence of various concentrations of crocin (0–100 μM) for 4, 24, 48 and 72 h and then subjected to MTT cytotoxicity assay. Crocin showed inhibitory effects on the cell growth rate of A2780 cells in a concentration and time-dependent manner (Fig. 1a). Crocin exhibited a similar inhibitory pattern with less potency in A2780/RCIS (Fig. 1b), in which treatment with 60–100 μM and 80–100 μM of crocin significantly reduced the survival of A2780 and A2780/RCIS, respectively (*P* < 0.05 vs. control).

### Expression of MRP1 and MRP2 in A2780 cell lines

**Fig. 2:**
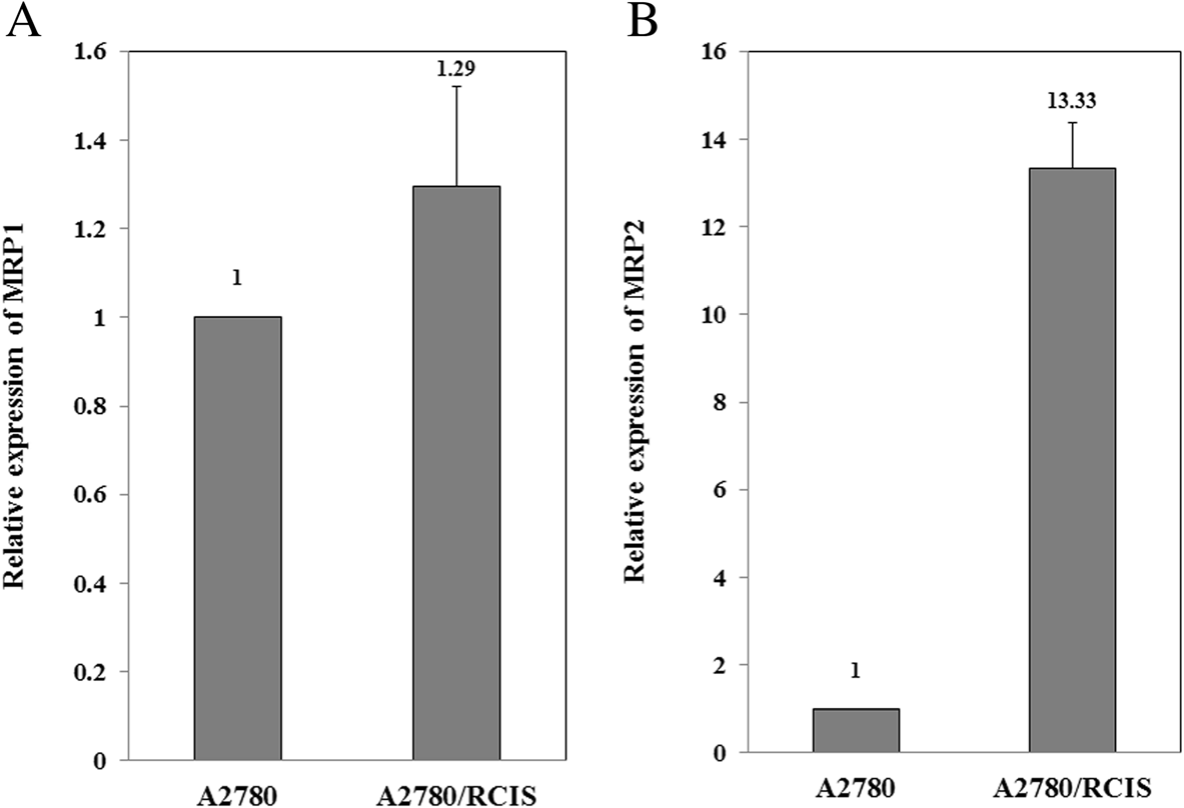
Basal expressions of MRP1 (**a**) and MRP2 (**b**) mRNA in ovarian cancer cell lines were studied by real-time RT-PCR. The MRP mRNA level is compared with the drug-resistant cell line A2780/RCIS and parental drug-sensitive cell line A2780. Real-time RT-PCR analysis was performed on total RNA extracted from cells. Values were normalized to the β-actin content of samples and expressed as mean ± SD (*n* = 3).

Real-time RT-PCR was used to assess the basic level of the mRNA expression of MRP1 and MRP2 in cisplatin-resistant A2780/RCIS cells and sensitive parental A2780 cell line. As shown in Fig. 2a, the MRP1 mRNA level in the A2780/RCIS cell line was 1.29 times more than its expression level in A2780 cells. Also, the results showed that the expression level of MRP2 mRNA in the resistant cell line A2780/RCIS was about 13 times more than the MRP2 mRNA level in parental A2780 cells (Fig. 2b).

### Effect of crocin on MRP1 and MRP2 gene expression

**Fig. 3:**
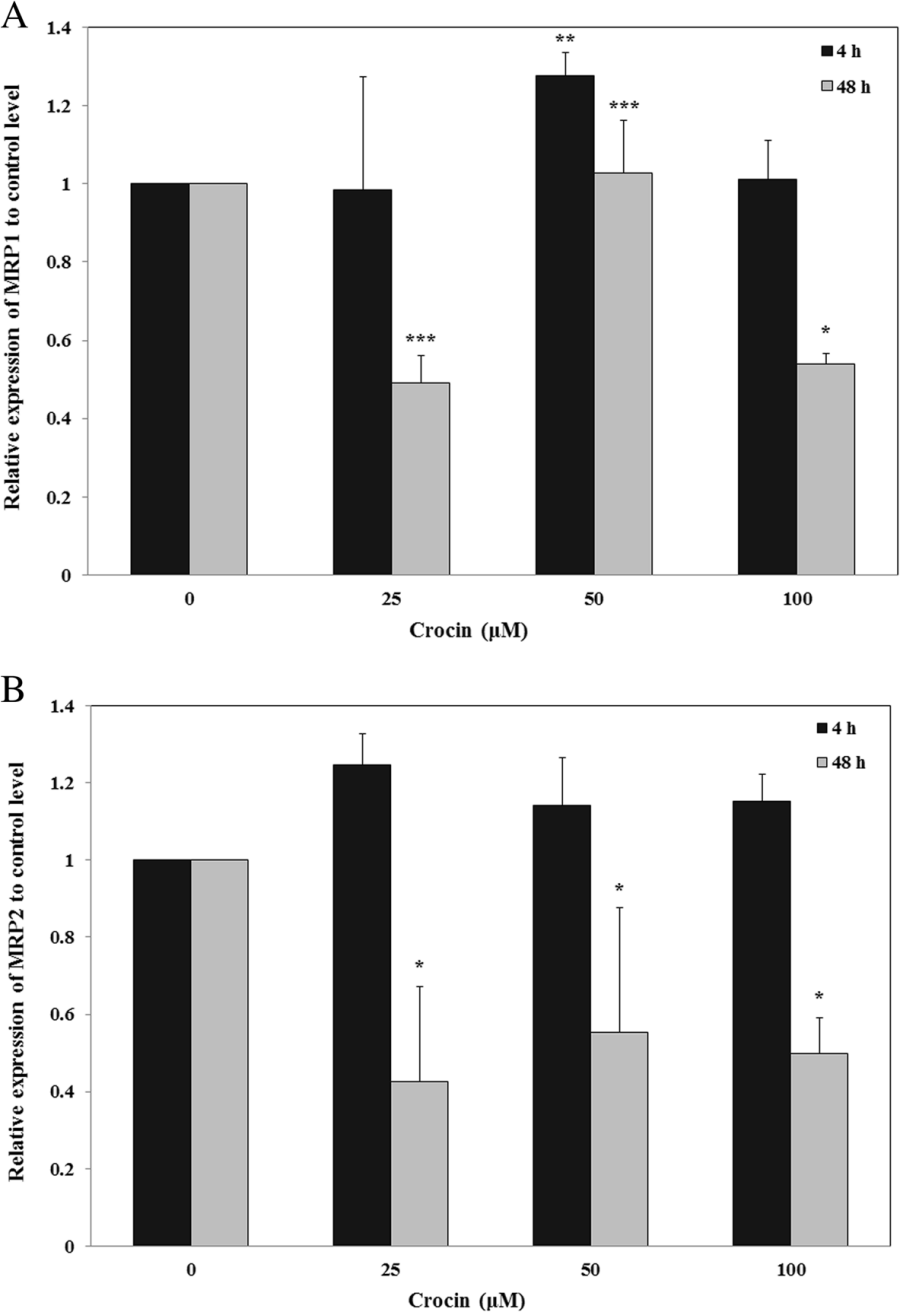
The effects of crocin on the levels of MRP1 (**a**) and (**b**) mRNA in the A2780/RCIS cell line. Cells were treated for 4 and 48 h with crocin (0–100 μM), and MRP1 and MRP2 mRNA expressions were measured by real-time RT-PCR using total RNA extracted from control and treated cells. Values were normalized to the β-actin content of the samples. The results were expressed as the target/reference ratio of the treated samples divided by the target/reference ratio of the untreated control sample and expressed as mean ± SD (*n* = 3); *, *p* < 0.05; **, *p* < 0.01; ***, *p* < 0.001

To evaluate the expression of MRP1 and MRP2 mRNA after treatment with crocin, the cells were treated with different concentrations of crocin (0–100 μM) for 4 and 48 h. Expression of MRP transporters was studied using Real-time RT-PCR. The results showed that crocin significantly reduced the expression level of MRP1 (up to about 50 %) in A2780/RCIS cells at 48 h (Fig. 3a). Similarly, crocin could significantly reduce MRP2 mRNA expression (up to 60 %) in cisplatin-resistant A2780/RCIS cells in a time dependent manner in compared with the control level (Fig. 3b).

### Effect of crocin on multi-drug resistance protein activity

**Fig. 4:**
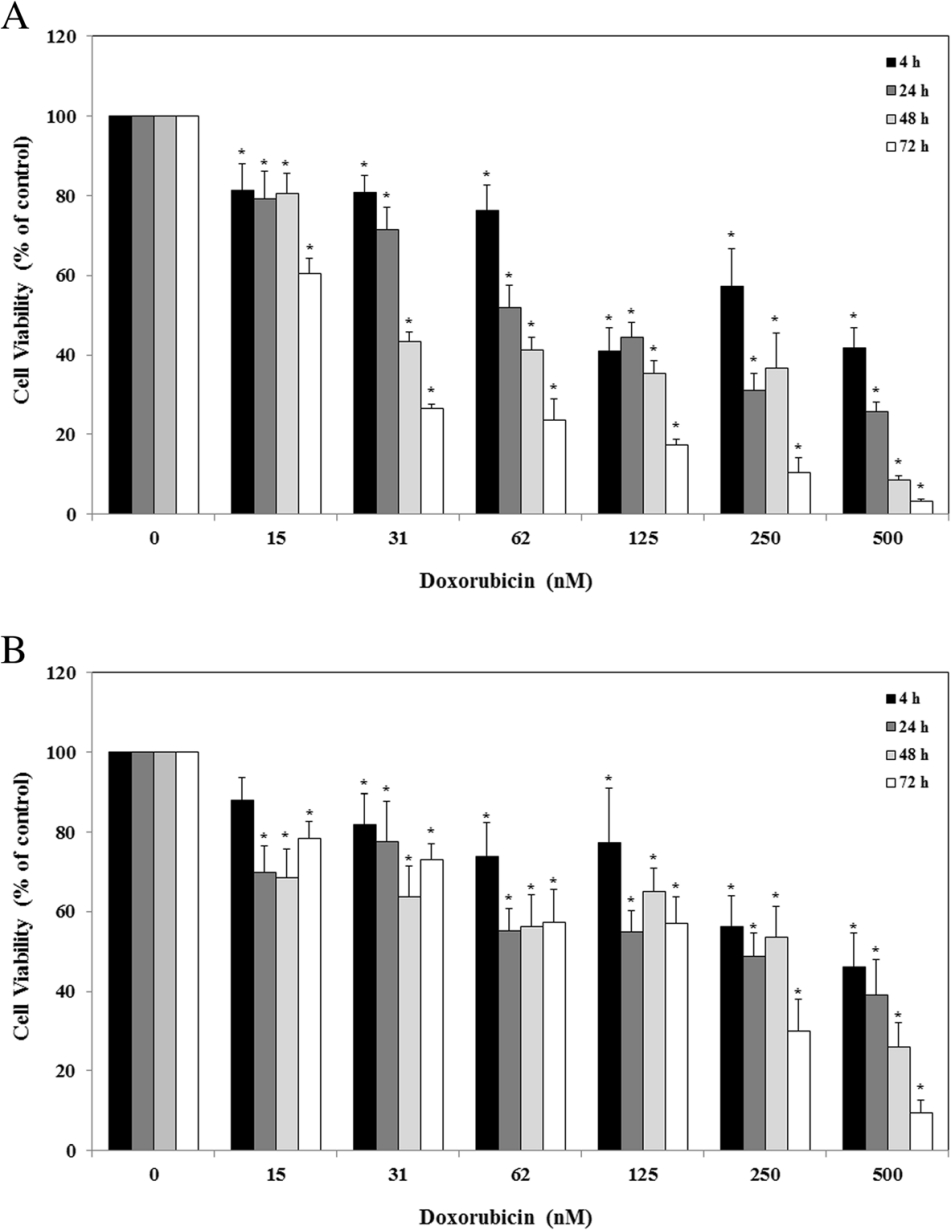
The effects of doxorubicin on cell viability of A2780 (**a**) and A2780/RCIS (**b**) cell lines. The cells were incubated with different concentrations of doxorubicin (0–500 nM) for 4, 24, 48 and 72 h. Cell viability was measured by the MTT assay. Each experiment was repeated independently three times in triplicate tests and data are shown as mean ± SD. *P ≤ 0.001

In order to evaluate the effects of crocin on MRP transporters activity, sensitivity of tested cell lines to doxorubicin in the presence or absence of crocin were studied. At first it was necessary to find the IC_50_ of doxorubicin on A2780 and A2780/RCIS cells. For this purpose, cells were treated with different concentrations of doxorubicin (0–500 nM) for 4, 24, 48, and 72 h. Doxorubicin significantly reduced the proliferation rate of A2780 (Fig. 4a) and A2780/RCIS cells (Fig. 4b) in a concentration and time-dependent manner. These anti-proliferative effects of doxorubicin were with a higher intensity in the parental cisplatin-sensitive cell line A2780 in comparison with the resistant cell line A2780/RCIS.

To test the combining effects of crocin and doxorubicin on the cell survival of A2780 cell lines, 30 different combinations (0–100 μM of crocin with 0–500 nM of doxorubicin) were evaluated using MTT assay. The combinatorial effects on cell survival were analyzed after 4, 24, 48, and 72 h incubation. There were no significant differences in A2780 cell viabilities between all crocin + doxorubicin concentrations and controls with the same amount of doxorubicin (data not shown). On the other hand, different concentrations of crocin could increase the percentage of doxorubicin cytotoxicity in A2780/RCIS, almost all in a time and concentration-dependent manner (Table 1). While crocin could not change the A2780/RCIS cell sensitivity to doxorubicin in 4 h, there were significant differences between the IC_50_ of samples that were treated with crocin + doxorubicin and IC_50_ of controls that were with the same amount of doxorubicin after 48 h (144 nM vs. 349 nM, *P* < 0.001) and 72 (162 nM vs. 326 nm, *P* < 0.01).

**Table 1:** The effects of different concentrations of crocin on the cell survival percentages of A2780/RCIS cell lines under treatment with doxorubicin (mean ± SEM)

Time (h)	Dox (nM)	Crocin (μM)			
		0	25	50	100
4 h	16	97.04 ± 2.18	97.39 ± 10.06	95.88 ± 3.49	98.77 ± 3.05
	62	92.19 ± 4.94	96.51 ± 2.36	92.57 ± 0.70	95.91 ± 1.34
	125	91.52 ± 3.92	95.35 ± 0.91	91.77 ± 4.48	92.74 ± 2.33
	250	93.56 ± 2.70	95.13 ± 4.03	83.37 ± 5.05	84.1 ± 2.48
	500	77.64 ± 4.70	91.01 ± 9.06	80.95 ± 4.23	86.59 ± 3.93
24 h	16	95.54 ± 2.16	88.37 ± 1.55	96.30 ± 1.78	87.02 ± 6.36
	62	96.98 ± 2.80	95.83 ± 2.48	96.94 ± 1.63	93.58 ± 1.41
	125	96.95 ± 1.31	92.23 ± 0.51	96.33 ± 3.51	75.37 ± 5.73
	250	94.75 ± 2.33	75.84 ± 4.77	51.68 ± 4.70^***^	73.80 ± 5.33
	500	51.08 ± 1.17	26.58 ± 4.38^***^	35.35 ± 8.88^***^	23.06 ± 7.58^***^
48 h	16	99.39 ± 2.57	85.17 ± 3.68	80.87 ± 5.94^*^	53.66 ± 8.40^***^
	62	95.07 ± 3.99	73.01 ± 3.41^***^	60.60 ± 2.85^***^	54.96 ± 4.94^***^
	125	83.10 ± 2.02	71.49 ± 2.09	31.12 ± 8.98^***^	44.45 ± 6.49^***^
	250	53.04 ± 1.34	53.86 ± 1.73	25.77 ± 5.78^***^	7.219 ± 0.14^***^
	500	32.68 ± 3.40	19.11 ± 1.95	8.149 ± 3.16^***^	3.85 ± 0.29^***^
72 h	16	96.68 ± 3.55	84.31 ± 2.84	63.00 ± 1.68	63.29 ± 3.29^**^
	62	79.20 ± 3.88	65.62 ± 2.16	54.11 ± 5.12^***^	55.52 ± 1.85^***^
	125	60.52 ± 2.88	53.58 ± 1.63	47.77 ± 4.52^***^	34.41 ± 3.53^***^
	250	50.13 ± 2.80	33.17 ± 2.96^**^	34.49 ± 3.05^***^	28.09 ± 2.30^***^
	500	36.55 ± 2.82	22.21 ± 1.16	22.66 ± 1.71	9.27 ± 0.93^***^

*Note*: The results of the LSD test, which compared the effects of all crocin concentrations on the toxicity of each of the various concentrations of doxorubicin during 4, 24, 48, and 72 h. *, *p* < 0.05; **, *p* < 0.01; ***, *p* < 0.001

## Discussion

Multidrug resistance (MDR) is one of the most important reasons for the insufficient effectiveness of chemotherapy drugs in cancer treatment. A major mechanism involved in MDR, is the presence of some of the ATP-binding cassette transporters (ABC) like Multidrug resistance-associated proteins 1 and 2 (MRP1 and MRP2), which are expressed on the surface of cells and pumps chemotherapy drugs out of the cell. In recent years, scientists have tried to find efficient inhibitors of special ABC transporters to overcome MDR phenomenon [27]. Crocin, a major constitute of saffron [10], has shown anticancer activities against several cancer cell lines [19–23], and could be used clinically for the prevention and treatment of cancer in the near future. In this study, we evaluated the effects of crocin on the expression and function of MRP1 and MRP2 in the human ovarian carcinoma cell line A2780 and MRP2-overexpressing cell line A2780/RCIS.

It has been previously shown that induction of the MRP2 expression in human cancer cell lines enhances the resistance to doxorubicin [28], while down regulation of MRP2 enhances cell sensitivity to doxorubicin [29]. Based on these studies and similar studies for MRP1, doxorubicin has been introduced as a substrate of MRP1 and MRP2 transporters [30]. The results of the present study have shown that the MRP1 and MRP2 expression level in the cisplatin-resistant A2780/RCIS cell line was 1.29 and 13 times more than its expression level in its parental sensitive cell line A2780, respectively. On the other hand, doxorubicin had anti-proliferative effects on the A2780 cells with higher intensity when compared with the A2780/RCIS cell line. These results indicated that the higher expression of MRP transporters in A2780/RCIS was accountable for the higher efflux of doxorubicin that resulted to its lower intracellular concentration and lower anti-proliferative activity in this drug-resistant cell line. Interestingly, crocin has shown a similar inhibitory pattern on cell growth of tested cell lines in which its anti-proliferative activity had less potency in A2780/RCIS in compared with the parental sensitive cell line. Totally, these results suggest that crocin could be a substrate of MRP transporters.

Several studies have investigated the molecular mechanism of anticancer activities of crocin against different human cancer cell lines. In one study, the differentially expressed gene of the bladder cancer T24 cell line after treatment with or without crocin has been evaluated using the cDNA microarray. The results showed that under crocin treatment, 836 genes were up-regulated or down-regulated, which were replication factor or were involved in cell cycle controlling and DNA cell apoptosis [31]. Similarly, other studies have shown that crocin could arrest the tumor cell cycle and induce apoptosis by inhibition of the expression of Bcl-2, survivin, cyclin D1, and lactate dehydrogenase A (LDHA) or by up-regulation the expression and activity of Bax and nuclear factor erythroid 2-related factor 2 (Nrf2), and by inhibition of the telomerase activity [10, 19, 20, 32, 33]. The investigation of cellular targets of crocin using proteomic screening showed that crocin physically binds to a wide range of cellular proteins such as membrane transporters and enzymes involved in ATP and redox homeostasis [34]. In another study, researchers investigated the inhibition ability of a selection of carotenoids including β-carotene, crocin, retinoic acid, canthaxanthin, and fucoxanthin on the P-glycoprotein (P-gp; MDR1). These carotenoids decreased P-gp mRNA expression levels; increased accumulation of cytotoxic agents which are P-gp substrates that cause to enhance their cytotoxicity effects. Totally, they concluded that carotenoids could be used as adjuvants which are chemosensitizer in chemotherapy [35].

In this study, we aimed to evaluate the effects of crocin on MRP transporter expression and function. For this purpose the sensitivity of tested cell lines to doxorubicin in the presence or absence of non-toxic concentrations of crocin were studied. The MRP activity assay has been designed in two ways, short time exposure with crocin to evaluate the direct interaction between crocin and existing active transporters, and longtime exposure with crocin to evaluate the indirect effect of crocin on the transporter activity as a result of its modifications on the expression level. Interestingly, crocin could not change the A2780/RCIS cell sensitivity to doxorubicin in 4 h, while it significantly increased doxorubicin cytotoxicity after 48 and 72 h in a concentration-dependent manner in the drug-resistant A2780/RCIS cell line, but not in parental drug-sensitive A2780 cells. On the other hand, the real-time PCR results showed that crocin significantly reduced the mRNA expression level of MRP1 and MRP2 in A2780/RCIS cells. Totally, these results indicated that crocin could suppress drug resistance via down regulation of MRP gene expression in the human ovarian cancer resistance cell line.

## Conclusion

In this study we aimed to investigate the ability of crocin on inhibiting multidrug resistance in human ovarian cancer cells by interfering with MRP 1 and MRP2 transporters. The results showed that crocin could decrease the gene expression of MRP1 and MRP2 and exert MDR reversal, and enhance the cytotoxicity of doxorubicin in the human MRP2 overexpressing cell line A2780/RCIS. This study suggests that the application of crocin in combination with chemotherapeutics in cancer treatment could be an effective method to improve the efficacy of chemotherapy and moderate the impact of MDR.

## Abbreviations

ABC, ATP-binding Cassette Transporters; DMSO, Dimethyl Sulfoxide; FBS, Fetal Bovine Serum; LDHA, Lactate Dehydrogenase A; LPS, Lipopolysaccharides; MDR, Multidrug Resistance; MRP, Multidrug Resistance-associated Protein; MTT, 3-(4, 5-dimethylthiazol-2-yl)-2, 5-diphenyl tetrazolium bromide; Nrf2, Nuclear Erythroid 2-related Factor 2; RCIS, Cisplatin-Resistant Derivative; Real-time RT-PCR, Real-rime Reverse Transcription Polymerase Chain Reaction; ROS, Reactive Oxygen Species; PBS, Phosphate-Buffered Saline; P-gp, P-glycoprotein
